# Association of Uncommon, Noncoding Variants in the *APOE* Region With Risk of Alzheimer Disease in Adults of European Ancestry

**DOI:** 10.1001/jamanetworkopen.2020.17666

**Published:** 2020-10-22

**Authors:** Elizabeth E. Blue, Anqi Cheng, Sunny Chen, Chang-En Yu

**Affiliations:** 1 Division of Medical Genetics, Department of Medicine, University of Washington School of Medicine, Seattle; 2 Department of Biostatistics, University of Washington, Seattle; 3 Geriatric Research, Education, and Clinical Center, Veterans Affairs Puget Sound Health Care System, Seattle, Washington; 4 Division of Gerontology and Geriatric Medicine, Department of Medicine, University of Washington, Seattle

## Abstract

**Importance:**

The ε2 and ε4 alleles of the apolipoprotein E (*APOE*) gene are associated with Alzheimer disease (AD) risk. Although nearby genetic variants have also been shown to be associated with AD, including rs2075650 in the *TOMM40* gene and rs4420638 near the *APOC1* gene, it is unknown whether these associations are independent of the ε2 and ε4 alleles.

**Objective:**

To assess whether variants near *APOE* are associated with AD independently of the ε2/ε3/ε4 genotype.

**Design, Setting, and Participants:**

In this genetic association study of the Alzheimer’s Disease Genetics Consortium imputed genotype at data, 14 415 variants near *APOE* (±500 kilobase) for 18 795 individuals with European ancestry were tested for association with AD using 4 logistic mixed models adjusting for sex, cohort, population structure, and relatedness. Model 1 had no *APOE* adjustment, and model 2 adjusted for the count of ε2 and ε4 alleles. Model 3 was restricted to ε3 homozygotes, and model 4 was restricted to ε4 homozygotes. Data were downloaded from May 31, 2018, to June 3, 2018, and analyzed from November 1, 2018, to June 24, 2020.

**Main Outcomes and Measures:**

Alzheimer disease affectation status was defined by clinicians using standard National Institute of Neurological and Communicative Disorders and Stroke and Alzheimer Disease and Related Disorders Association criteria. Association was evaluated using Score tests; results with *P* < .05 divided by the number of independent tests per model were considered statistically significant.

**Results:**

Among the 18 795 individuals in the study, 9704 were affected by AD and 9066 were control individuals; the median age at onset/evaluation was 76 (interquartile range, 70-82) years; and 11 167 were female (59.4%). Associations with AD were found for rs2075650 (odds ratio [OR], 2.59; 95% CI, 2.45-2.75; *P* = 3.19 × 10^−228^) and rs4420638 (OR, 2.77; 95% CI, 2.62-2.94; *P* = 2.99 × 10^−254^) without *APOE* adjustment. Although rs2075650 was nominally associated with AD among the ε4 homozygotes (OR, 1.33; 95% CI, 1.00-1.77; *P* = .047), the association between rs4420638 and AD was eliminated by *APOE* adjustment (model 2 OR, 1.06 [95% CI, 0.96-1.18; *P* = .24]; model 3 OR, 1.13 [95% CI, 0.95-1.34; *P* = .18]; model 4 OR, 0.90 [95% CI, 0.56-1.45; *P* = .66]). There was a significant association between rs192879175 and AD among ε3 homozygotes (OR, 0.50; 95% CI, 0.37-0.68; *P* = 8.30 × 10^−6^).

**Conclusions and Relevance:**

The results of this genetic association study suggest that ε2/ε3/ε4 alleles are not the only variants in the *APOE* region that are associated with AD risk. Additional work with independent data is needed to replicate these results.

## Key Points

**Question:**

Is genetic variation near the apolipoprotein E gene (*APOE*) associated with risk of Alzheimer disease (AD) independently of the ε2/ε3/ε4 genotype?

**Findings:**

In this genetic association study of 18 795 participants of European ancestry from the Alzheimer’s Disease Genetics Consortium, an association was found between rs2075650 and AD risk among ε4 homozygotes and a significant association was found between rs192879175 and AD risk among ε3 homozygotes.

**Meaning:**

These findings suggest that even among individuals with the same ε2/ε3/ε4 genotype, genetic variation within the *APOE* neighboring region may be associated with risk of AD.

## Teaser

This genetic association study assesses whether variants near the apolipoprotein E gene (*APOE*) are associated with Alzheimer disease independently of ε2/ε3/ε4 genotype.

## Introduction

The association between the apolipoprotein E (*APOE* [OMIM 107741]) gene and Alzheimer disease (AD) has been known for longer than 25 years^1,2^ and has remained the strongest and most consistent association between AD risk and a common DNA variant.^3,4^ Dozens of genetic loci are associated with risk of AD, and hundreds of variants across 3 genes (*APP* [OMIM 605714], *PSEN1* [OMIM 104311], and *PSEN2* [OMIM 600759]) are known to cause early-onset, autosomal dominant forms of AD.^4,5,6^ This genetic heterogeneity has also been observed at the *APOE* locus. Two independent missense variants in *APOE*, rs429358 and rs7412, are consistently associated with large effects on AD risk, and together define the ε2/ε3/ε4 alleles. Associations between many other single-nucleotide variants (SNVs) at the *APOE* locus with AD risk, age at onset, and/or biomarkers have been reported.^7,8^

Whether the association between SNVs in the *APOE* region and AD is independent of the effects of rs429358 and rs7412 is not settled. Many of these SNVs are in linkage disequilibrium (LD) with rs429358 in European ancestry samples, and most are noncoding changes that could affect gene expression.^7,8^ The *APOE* locus includes a long cluster of genes transcribed in the same direction, suggesting that they may be coregulated by *cis* regulatory elements. These genes have also been implicated in shared biological pathways, including lipid metabolism, the immune system, and mitochondrial function,^5,7^ which suggests that changes in either quality or quantity of the products of these genes may also be associated with AD.

Two noncoding SNVs at the *APOE* locus have consistently shown an association with AD risk and related traits: rs2075650 (the *TOMM40* SNV [OMIM 608061]) and rs4420638 (the *APOC1* SNV [OMIM 107710]). The association between these SNVs and AD is not always robust to *APOE* adjustment.^9,10^ Both SNVs are also associated with memory and cognitive function, cerebral spinal fluid biomarkers for immune response,^11^ oxidative stress markers,^9^ and longevity.^12,13,14^ However, because both SNVs are in moderate LD (0.2 < *r*^2^ < 0.8) with rs429358, these associations may not be independent of the ε4 allele.

We investigated whether rs2075650, rs44209638, or other SNVs in the extended *APOE* locus are associated with risk of AD independently of ε2/ε3/ε4 genotype in a large cohort with European ancestry. We hypothesized that the analytical strategy to adjust for *APOE* effects may influence these association signals.

## Methods

### Samples and Genotype Data

This genetic association study used Alzheimer’s Disease Genetics Consortium (ADGC) data, which were accessed through an application on the ADGC website.^15^ All participants reported European ancestry. This study was approved by the University of Washington institutional review board and followed the Strengthening the Reporting of Genetic Association Studies (STREGA) reporting guideline. This study evaluated publicly available deidentified data provided by the ADGC. Informed consent was obtained for all research participants as previously described.^16^

The ADGC imputed genotype data were previously generated using the segmented haplotype estimation and imputation tool (SHAPEIT)^17^ and IMPUTE, version 2,^18^ or MaCH^19^ and Minimac^20^ software and the 1000 Genomes Project (1KGP) sequence data as reference (phase 3; hg19/GRCh37),^5,21^ in which imputed variants with minor allele frequencies (MAFs) of at least 0.01 and either an *r*^2^ or an information measure of less than 0.40 were removed. After excluding 2 data sets owing to incomplete data files, we extracted the SNVs on a bead chip array (Infinium OmniExpress; Illumina) to create a genome-wide association study (GWAS) panel used to estimate principal components, relatedness, and genomic inflation (λ statistic).^22^ Single-nucleotide variants with an MAF of less than 0.05, variant-level missing rate of greater than 0.05, or ambiguous alleles were excluded from analysis, as were samples with individual-level missing rate of greater than 0.05; 510 665 variants in 18 795 participants remained. We extracted the 14 415 imputed SNVs within the *APOE* gene (±500 kilobase [kb]) (chromosome 19: 44 909 039-45 912 650) for association testing. Case individuals were defined as those affected by AD as determined by clinicians using the National Institute of Neurological and Communicative Disorders and Stroke and Alzheimer Disease and Related Disorders Association criteria,^23,24^ and control individuals were those not affected by AD. *APOE* genotypes (ε2/ε3/ε4) were extracted from the cohort-specific covariate files. *APOE* was genotyped differently across ADGC cohorts.^16^

### Statistical Analysis

Data were downloaded from May 31, 2018, to June 3, 2018, and analyzed from November 1, 2018, to June 24, 2020. The GENESIS package was used to test for the association between SNVs and AD risk,^25,26^ an approach that accounts for both population and pedigree structure (eMethods in the Supplement). PC-AiR^27^ performed a principal components analysis on the GWAS panel to detect population structure, accounting for kinship estimates provided using the KING approach for robust inference.^28^ PC-Relate^29^ then used these principal components to estimate a genetic relatedness matrix that is adjusted for population structure. Plots of the first 2 principal components were used to identify outliers among those with self-reported European ancestry. We fit 4 logistic mixed models adjusted for sex, cohort, the first 10 principal components, and a polygenic random effect with covariance structure given by the genetic relatedness matrix. Model 1 included all samples with no *APOE* adjustment, model 2 included all samples and adjusted for ε2 and ε4 allele counts, model 3 was restricted to ε3 homozygotes, and model 4 was restricted to ε4 homozygotes. Score tests were performed for each logistic model for all SNVs with an MAF of greater than 0.01, with missing genotype data imputed using observed allele frequencies within the data. We estimated the odds ratio (OR) and its 95% CI as follows: OR = Exp × (score statistic/standard error^2^) and 95% CI = ±1.96 × (1/standard error). For each model *m*, the number of independent tests *t_m_* was estimated using the genetic type 1 error calculator.^30^ Statistical significance was defined as *P* < .05/*t_m_*. Linkage disequilibrium between pairs of SNVs was measured using PLINK, version 1.07.^31^ Correlations between ε2 and ε4 genotypes and imputed genotypes at rs7412 and rs429358 were estimated using R, version 3.5.2.^32^ The mismatch between observed and expected ε2 and ε4 genotypes was calculated as the number of alleles differing between the observed and imputed genotypes divided by the number of alleles observed. Basic variant annotations, including LD in the 1KGP subset with European ancestry, were performed using HaploReg, version 4.1.^33^ Ancestry-matched reference allele frequencies (European MAF) were extracted for non-Finnish Europeans in the gnomAD database, version 2.1.^34^

## Results

### Summary Statistics

The data within the *APOE* region includes 14 415 SNVs and 18 795 individuals, of whom 11 167 were women (59.4%) and 7628 were men (40.6%) (median age at onset/evaluation, 76 [interquartile range, 70-82] years); 9704 were affected by AD (51.6%), and 9066 were controls (51.6%) (eTable 1 in the Supplement). Among cases, the ε2 allele frequency was 680 of 19 408 (3.5%), and the ε4 allele frequency was 7360 of 19 408 (37.9%); among controls, the ε2 frequency was 1444 of 18 132 (8.0%) and the ε4 frequency was 2490 of 18 132 (13.7%). We observed 71 ε2 homozygotes, 8848 ε3 homozygotes, and 1503 ε4 homozygotes. No outliers were identified by principal components analysis (eFigure 1 in the Supplement), and relatedness estimates were robust to the inclusion of genotypes from chromosome 19 (eFigure 2 in the Supplement). The number of independent tests within the *APOE* region was similar across analysis models (*t_1_* and *t_2_*, 1128; *t_3_*, 1055; and *t_4_*, 1013), with similar significance thresholds (*t_1_* and *t_2_*, *P* = 4.43 × 10^−5^; *t_3_*, *P* = 4.74 × 10^−5^; and *t_4_*, *P* = 4.94 × 10^−5^).

### Associations of rs2075650 and rs4420638 With ε2, ε4, and AD Risk

There was a stronger LD among rs2075650 (*TOMM40*), rs4420638 (*APOC1*), and rs429358 (ε4) in the ADGC data than in 1KGP Europeans, and none of these SNVs were in LD with rs7412 (ε2) (eTable 2 in the Supplement). Among the 1KGP Europeans, both rs2075650 (*r*^2^ = 0.48) and rs4420638 (*r*^2^ = 0.65) had moderate LD with rs429358 and modest LD with each other (*r*^2^ = 0.30). These correlations were strengthened in the ADGC data, in which *r*^2^ ranged from 0.50 to 0.83 among these 3 SNVs.

The association between AD status and the *TOMM40* and *APOC1* SNVs varied across models (Table 1), each showing no evidence for genomic inflation (λ_1_ = 1.03; λ_2_ = 1.03; λ_3_ = 1.01; and λ_4_ = 0.99) (eFigure 3 in the Supplement).

**Table 1.** Association Between the *TOMM40*, *APOC1*, and *APOE* SNVs and AD With and Without *APOE* Adjustment or Stratification

Model^a^	SNV	Nearest gene	No. of Participants	AAC	AAF	OR (95% CI)	*P* value
1	rs2075650	*TOMM40*	18 211	8108	0.2226	2.59 (2.45-2.75)	3.19 × 10^−228^^b^
2	rs2075650	*TOMM40*	18 211	8108	0.2226	1.09 (0.99-1.19)	.07
3	rs2075650	*TOMM40*	8642	746	0.0432	1.16 (0.98-1.38)	.09
4	rs2075650	*TOMM40*	1426	2106	0.7400	1.33 (1.00-1.77)	.047^c^
1	rs4420638	*APOC1*	15 894	7967	0.2506	2.77 (2.62-2.94)	2.99 × 10^−254^^b^
2	rs4420638	*APOC1*	15 894	7967	0.2506	1.06 (0.96-1.18)	.24
3	rs4420638	*APOC1*	7821	674	0.0431	1.13 (0.95-1.34)	.18
4	rs4420638	*APOC1*	1058	1893	0.8900	0.90 (0.56-1.45)	.66

Abbreviations: AAC, alternate allele count; AAF, alternate allele frequency; AD, Alzheimer disease; *APOE*, apolipoprotein E; OR, odds ratio; SNV, single-nucleotide variant.

^a^ Model 1 included all samples, no *APOE* adjustment; model 2, all samples, adjusted for *APOE* ε2 and ε4 allele counts; model 3, restricted to ε3 homozygotes; and model 4, restricted to ε4 homozygotes.

^b^ Indicates passing the model-specific significance threshold.

^c^ Indicates nominally significant.

Each SNV was significantly associated with AD without *APOE* adjustment (model 1) (OR for rs2075650, 2.59 [95% CI, 2.45-2.75; *P* = 3.19 × 10^−228^]; OR for rs4420638, 2.77 [95% CI, 2.62-2.94; *P* = 2.99 × 10^−254^]), although these associations weakened with *APOE* adjustment or stratification. rs4420638 was not associated with AD with *APOE* adjustment (model 2: OR, 1.06; 95% CI, 0.96-1.18; *P* = .24), among ε3 homozygotes (model 3: OR, 1.13; 95% CI, 0.95-1.34; *P* = .18), or among ε4 homozygotes (model 4: OR, 0.90; 95% CI, 0.56-1.45; *P* = .66). The association between rs2075650 and AD was nominally significant among ε4 homozygotes (model 4) (OR, 1.33; 95% CI, 1.00-1.77; *P* = .047) but failed to reach significance after *APOE* adjustment (model 2; OR, 1.09; 95% CI, 0.99-1.19; *P* = .07) or among ε3 homozygotes (model 3; OR, 1.16; 95% CI, 0.98-1.38; *P* = .09).

Another *TOMM40* variant (rs10524523, also known as poly-T 523) has been reported to be associated with AD risk^35^ but was not available in ADGC data. Using a proxy SNV, rs8106922, which best defines the phylogenetic clade separating long vs short poly-T alleles,^36^ we found that rs2075650, rs4420638, rs429358, and rs7412 were not in LD with rs8106922 in ADGC data or 1KGP Europeans (*r*^2^ < 0.20). Although the minor allele at rs8106922 was significantly associated with reduced risk of AD under model 1 (OR, 0.69; 95% CI, 0.65-0.72; *P* < .001), the association was not significant under any model adjusting for or stratifying by *APOE* genotype (eTable 3 in the Supplement).

### Imputed vs Measured *APOE* Genotyping

We observed discordance between the observed ε2 and ε4 genotypes and the imputed genotypes at the SNVs used to define them. Both rs429358 (ε4) and rs7412 (ε2) were polymorphic in the imputed data in which they should not have been observed, that is, among ε3 homozygotes (173 of 17 276 and 79 of 17 052 alleles, respectively) and ε4 homozygotes (2314 of 2492 and 41 of 2664 alleles, respectively). Within the ADGC data, the ε2 and rs7412 genotypes were correlated (*r*^2^ = 0.77; *P* < .001), with a 1.3% mismatch between observed and imputed genotypes, and both the correlation (*r*^2^ = 0.88; *P* < .001) and mismatch (2.3%) between the ε4 and rs429358 genotypes were higher. Both the correlation between observed and imputed genotypes and the mismatch between them varied by *APOE* genotyping strategies (eTable 4 in the Supplement). The SNV-based genotyping had the highest correlation with imputed ε2 (*r*^2^ = 0.81) and ε4 (*r*^2^ = 0.90), and high-throughput sequencing had the lowest (*r*^2^ = 0.47 and *r*^2^ = 0.78, respectively). This discordance between observed and imputed ε2 and ε4 genotypes may have led to spurious associations with AD; there was a nominal association between imputed genotypes at rs429358 and AD after *APOE* adjustment (model 2 OR, 1.16; 95% CI, 1.00-1.34; *P* = .04) and among ε3 homozygotes (model 3 OR, 1.73; 95% CI, 1.26-2.38; *P* = 6.32 × 10^−4^). Imputation accuracy varies based on both the observed marker panel and the reference data set; older arrays performed worse with the 1KGP reference panel used by the ADGC (rs7412, *r*^2^ = 0.75; rs429358, *r*^2^ = 0.82) than newer arrays (rs7412, *r*^2^ = 0.95; rs429358, *r*^2^ = 0.95), and both performed better when using the Haplotype Reference Consortium reference panel (*r*^2^ > 0.98).^37^

### Additional Associations of *APOE* Region SNVs and AD Risk

Among the 14 415 SNVs in the *APOE* region, we identified 1 significant association across models after correcting for the effective number of independent tests. The Figure provides Manhattan plots of the associations with AD across models 2 to 4, and Table 2 summarizes the 12 strongest associations with AD across these 3 models. One SNV (rs192879175) was significantly associated with AD among ε3 homozygotes (model 3 OR, 0.50; 95% CI, 0.37-0.68; *P* = 8.30 × 10^−6^). No other SNVs were significantly associated with AD after multiple testing correction. None of these 12 SNVs were common in the ADGC data set (MAF > 0.10) or in LD with either rs429358 or rs7412 (maximum *r*^2^ = 0.006), and the *BCAM* missense variant rs117737673 represented the only coding change.

**Figure.**
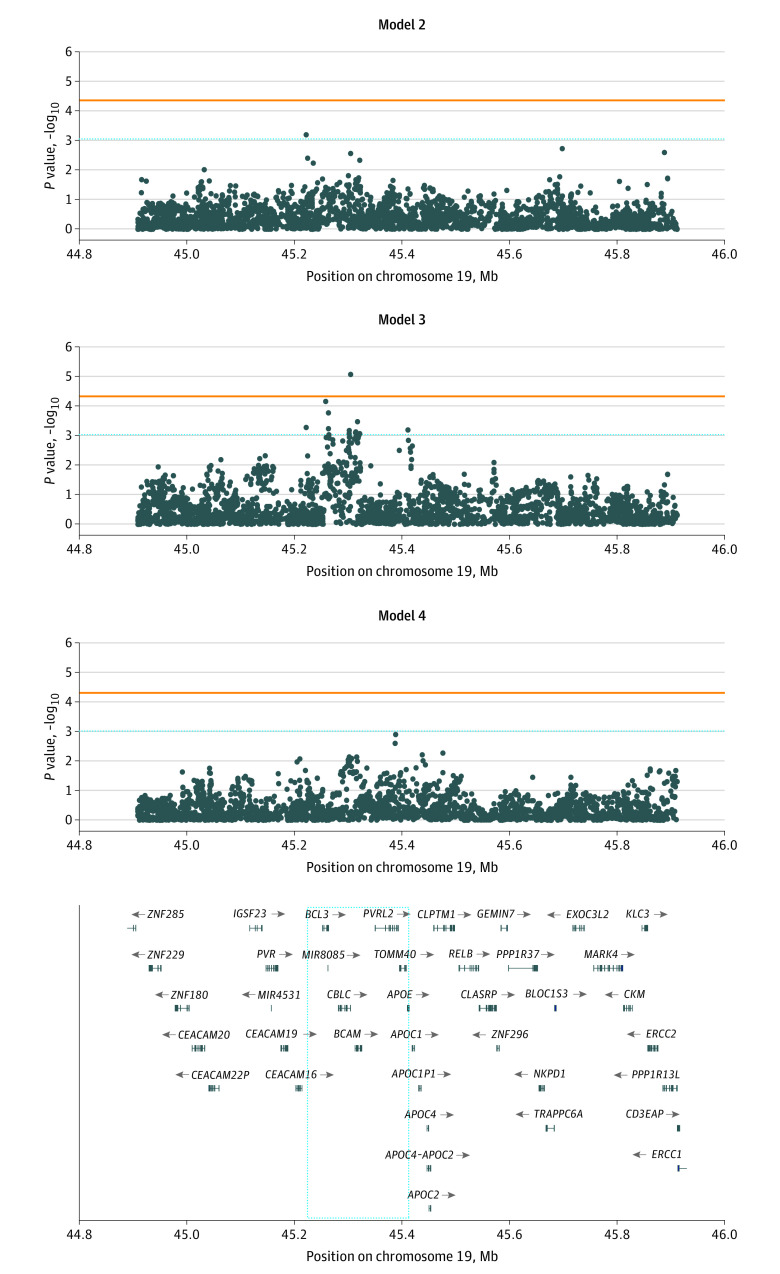
Manhattan Plot of Association Results Between Single-Nucleotide Variations in the Apolipoprotein E (*APOE*) Gene Region and Risk for Alzheimer Disease Across Analysis Models Variant positions on chromosome 19 are relative to the hg19/GRCh37 reference genome. For model 2, the analysis included all samples adjusted for *APOE* ε2 and ε4 allele counts, with 1128 effective independent tests. For model 3, the analysis was restricted to *APOE* ε3 homozygotes, with 1055 effective independent tests. For model 4, the analysis was restricted to *APOE* ε4 homozygotes, with 1013 effective independent tests. The horizontal orange line denotes the statistical significance threshold per model (*P* < .05/number of independent tests), whereas the blue dotted line denotes *P* = 1/number of independent tests. The blue dotted square highlights the genes falling within the region harboring variants with *P* < 1/effective number of tests. Mb indicates megabase.

**Table 2.** Additional SNVs Within the *APOE* Region With an Association With AD Status Across Models 2, 3, and 4

Model^a^	SNV	BP37	ALT	No. of participants	AAC	AAF	OR (95% CI)	*P* value
2	rs143764218	45222739	AC	16 714	915	0.03	0.76 (0.64-0.89)	6.26 × 10^−4^
3	rs143764218	45222739	AC	7794	507	0.03	0.69 (0.56-0.85)	5.20 × 10^−4^
3	rs1979377	45259002	C	7396	801	0.05	0.71 (0.59-0.84)	6.84 × 10^−5^
3	chr19:45264102:I	45264102	TG	7518	555	0.04	0.68 (0.56-0.83)	1.67 × 10^−4^
3	rs10416720	45264110	T	7491	846	0.06	0.75 (0.63-0.88)	5.72 × 10^−4^
3	rs145414981	45265003	C	7355	718	0.05	0.74 (0.62-0.88)	9.18 × 10^−4^
3	rs73572003	45302665	G	7982	1250	0.08	0.79 (0.69-0.91)	8.32 × 10^−4^
3	rs143695016	45302840	T	8003	1251	0.08	0.79 (0.68-0.90)	6.59 × 10^−4^
3	rs192879175	45305363	T	8635	256	0.01	0.50 (0.37-0.68)	8.30 × 10^−6^^b^
3	rs28399650	45314364	A	8633	433	0.03	0.68 (0.54-0.85)	7.80 × 10^−4^
3	rs28399652	45314975	G	8640	434	0.03	0.67 (0.54-0.85)	7.36 × 10^−4^
3	rs2968180	45318153	T	8218	1542	0.09	0.79 (0.70-0.90)	3.31 × 10^−4^
3	rs117737673	45322316	T	8489	546	0.03	0.70 (0.57-0.86)	8.42 × 10^−4^

Abbreviations: AAC, alternate allele count; AAF, alternate allele frequency; AD, Alzheimer disease; ALT, alternate allele; *APOE*, apolipoprotein E; BP37, position on the hg19 map; OR, odds ratio; SNV, single-nucleotide variant.

^a^ Model 2 included all samples, adjusted for *APOE* ε2 and ε4 allele counts; model 3, restricted to ε3 homozygotes; and model 4, restricted to ε4 homozygotes. The effective number of tests under model 2 was 1128 of 3408 SNVs; under model 3, 1055 of 3346 SNVs; and under model 4, 1013 of 3238 SNVs. All variants are on chromosome 19.

^b^ Indicates passing the model-specific significance threshold.

### Evidence for Replication

Limited evidence for replication of the significant associations presented in Tables 1 and 2 was available and was derived from 2 GWAS of AD in European ancestry samples. The family-based GWAS of the National Institute of Aging-Late Onset Alzheimer Disease Family Study (NIA-LOAD^38^) included association tests within *APOE* strata. That analysis of 1421 ε3 homozygotes did not provide evidence for an association between rs2968180 and AD, whereas the analysis of 408 ε4 homozygotes supported the association between rs2075650 and AD. This evidence was not independent of the ADGC, because the NIA-LOAD sample was represented in the ADGC LOAD cohort (eTable 1 in the Supplement). The stage 1 meta-analysis of the International Genomics Alzheimer Project data^39^ represented 53 711 participants, including 10 273 from the ADGC.^40^ We compared results from the International Genomics Alzheimer Project analysis of 34 152 ε4-negative participants with our analysis of ε3 homozygotes. Results were available for 4 SNVs from Table 2; the associations between AD and rs145414981 and rs1979377 were nominally significant, whereas the associations between AD and rs143695016 and rs73572003 were not.^39^

## Discussion

This study found an association between several SNVs for *APOE* and AD risk. Among these, rs192879175 was significantly associated with risk of AD among ε3 homozygotes, rs143764218 was nominally associated with AD after *APOE* adjustment and among ε3 homozygotes, and rs2075650 was nominally associated with AD among ε4 homozygotes. There was a stronger association between SNVs near *APOE* and AD status in the *APOE*-stratified vs the *APOE*-adjusted models. This finding was likely because these strata were restricted to individuals who shared 2 copies of an *APOE* allele identical by state and were therefore more likely to share recent common ancestry.

The *TOMM40* SNV rs2075650 has a long history of an association with AD and related traits, including 8 GWAS for AD risk^41,42,43,44,45,46,47,48^ and several studies of healthy aging and longevity.^12,13,49,50^ It is a common variant located within intron 2 of *TOMM40* (European MAF = 0.14). rs2075650 overlaps with promoter/enhancer histone marks in immune cells and brain tissues, is predicted to alter 8 transcription factor binding site motifs,^51^ and is significantly associated with *TOMM40*, *PVRL2,* and *HIF3A* expression levels.^11,52,53^ Both rs192879175 and rs143764218 are uncommon (rs192879175: European MAF, 0.01; rs143764218: European MAF, 0.05), are located between genes, and bear features consistent with regulatory variants. rs192879175 is 1.5 kb 3′ of *CBLC*, sits within enhancer histone marks and a DNase I hypersensitivity site in liver, and is predicted to alter a transcription factor binding site motif.^51^ Similarly, rs143764218 is 8.7 kb 3′ of *CEACAM16*, sits within promoter/enhancer histone marks and DNase I hypersensitivity site across multiple tissues, and is predicted to alter 7 transcription factor binding site motifs.^51^ Neither rs192879175 nor rs143764218 has previously been shown to be associated with AD or other traits by GWAS,^54^ perhaps owing to their uncommon allele frequencies.

We identified associations between noncoding variants in the *APOE* region and risk of AD. Haplotypic differences among participants sharing the same *APOE* genotype are associated with risk of AD.^55,56,57^ Haplotypes derived from rs429358, rs7412, and neighboring noncoding SNVs that vary in frequency across populations are associated with increased risk of AD.^55^ Admixture analyses in Puerto Rican, African American, and Caribbean Hispanic data sets have shown that ε4 alleles inherited on an African background are associated with reduced risk of AD compared with those inherited on a European background, again suggesting that haplotype structures correlated with ε4 vary between populations and are associated with AD risk.^56,57^ All SNVs with significant associations with AD were located within a 186-kb region immediately 5′ of *APOE*. All 5 genes in this region share the same transcriptional orientation as *APOE*, suggesting synchronized *cis* regulation might exist. Regulatory variants could modify this transcriptional pathway and subsequently change the gene expression profiles within this entire region.

Few AD genetics studies have accounted for *APOE* genotype, hampering replication efforts. As summarized above, 2 studies^38,39^ offered limited support for the 5 SNVs with evidence for association with AD in our study. However, both studies included a subset of the ADGC data analyzed herein and were not truly independent. Larger data sets with high-quality *APOE* genotype data are needed to replicate the results of the present study, particularly for the associations identified among ε4 homozygotes, including 1326 cases and 177 controls. Laboratory-based procedures such as molecular haplotyping, haplotype-based fine mapping,^58^ and reporter assays are needed to investigate the potential functional consequences of SNVs and how those consequences may influence AD pathogenesis.

### Limitations

This study has limitations. Imputed genotype data are not without error. Most of the discordant genotypes we observed involved ε2 or ε4 alleles being imputed as ε3 alleles, consistent with prior work^59^; this likely contributed to the spurious association between rs429358 and AD among the ε3 homozygotes. The ADGC data were collected on a mixture of older and newer arrays, which may explain some of the discordance we observed between the observed and imputed *APOE* genotypes. We observed lower mismatch rates at ε2 and ε4 among those genotyped by an SNV-based approach with high accuracy (error rate, 0.002^37^) compared with those genotyped by next-generation sequencing, suggesting that genotyping error may explain these differences. The stronger correlation between the *APOE* region genotypes in the ADGC compared with the 1KGP Europeans (consistent with previous reports of differing LD patterns between AD cases and controls^60^) suggests that using sequence data generated on a large and diverse sample set ascertained for AD status as a reference may improve the quality of imputed genotypes in AD GWAS. Our data represent only those with European ancestry; thus, our results may not apply to other populations.

## Conclusions

This genetic association study found that ε2/ε3/ε4 alleles as well as other variants in the *APOE* region were associated with AD risk. Although future work in independent data are needed to replicate these results, our findings appear to provide valuable new candidate sites for targeted genetic analyses on larger sample sets representing diverse ethnic groups. The findings suggest that increased LD between SNVs within the *APOE* region in samples ascertained for AD vs population samples may influence the accuracy of imputation within AD-related data sets. The correlation between imputed vs measured ε2 and ε4 genotypes within the ADGC varied by genotyping platform, suggesting next-generation sequencing at rs7412 and rs429358 may not be as accurate as alternative approaches. Association testing results in the *APOE* region varies between models adjusting for or stratifying by ε2/ε3/ε4 genotype; future GWAS using these alternative approaches may yield novel results in existing data sets.

## Notes

Supplement. **eMethods.** Software Tools **eTable 1.** Sample Summary Table by ADGC Cohort **eTable 2.** Evidence for Linkage Disequilibrium Among the *TOMM40*, *APOC1*, and *APOE* SNVs **eTable 3.** Evidence for Association Between rs8106922 With and Without *APOE* Adjustment or Stratification **eTable 4.** Comparison of *APOE* ε2 and ε4 Genotypes With Imputed Genotypes at rs7412 and rs429358 **eFigure 1.** Principal Components Analysis Results **eFigure 2.** Difference Between Kinship Estimates Based on Genotypes for All Autosomes vs All Autosomes Except Chromosome 19 **eFigure 3.** Quantile-Quantile Plots of Genome-Wide Association Tests Under Each Analysis Model **eReferences.**
