# Supplementary material for: Association of Uncommon, Noncoding Variants in the APOE Region With Risk of Alzheimer Disease in Adults of European Ancestry
Source: JAMA Netw Open. 2020 Oct 22;3(10):e2017666. doi: 10.1001/jamanetworkopen.2020.17666 (PMC7582128; doi:10.1001/jamanetworkopen.2020.17666)
Supplement: Supplement. — eMethods. Software Tools eTable 1. Sample Summary Table by ADGC Cohort eTable 2. Evidence for Linkage Disequilibrium Among the TOMM40, APOC1, and APOE SNVs eTable 3. Evidence for Association Between rs8106922 With and Without APOE Adjustment or Stratification eTable 4. Comparison of APOE ε2 and ε4 Genotypes With Imputed Genotypes at rs7412 and rs429358 eFigure 1. Principal Components Analysis Results eFigure 2. Difference Between Kinship Estimates Based on Genotypes for All Autosomes vs All Autosomes Except Chromosome 19 eFigure 3. Quantile-Quantile Plots of Genome-Wide Association Tests Under Each Analysis Model eReferences. [file jamanetwopen-e2017666-s001.pdf]

Supplementary Online Content

Blue EE, Cheng A, Chen S, Yu CE; Alzheimer's Disease Genetics Consortium. Association of uncommon, noncoding variants in the *APOE* region with risk of Alzheimer disease in adults of European ancestry. *JAMA Netw Open*. 2020;3(10):e2017666. doi:10.1001/jamanetworkopen.2020.17666

eMethods. Software Tools
eTable 1. Sample Summary Table by ADGC Cohort
eTable 2. Evidence for Linkage Disequilibrium Among the *TOMM40*, *APOC1*, and *APOE* SNVs
eTable 3. Evidence for Association Between rs8106922 With and Without *APOE* Adjustment or Stratification
eTable 4. Comparison of *APOE* ε2 and ε4 Genotypes With Imputed Genotypes at rs7412 and rs429358
eFigure 1. Principal Components Analysis Results
eFigure 2. Difference Between Kinship Estimates Based on Genotypes for All Autosomes vs All Autosomes Except Chromosome 19
eFigure 3. Quantile-Quantile Plots of Genome-Wide Association Tests Under Each Analysis Model
References.

This supplementary material has been provided by the authors to give readers additional information about their work.

©2020 Blue EE et al. *JAMA Network Open*.

eMethods. Software Tools

This section focuses exclusively on naming the specific tools used within the named software packages.

We estimated kinship using the KING-robust [1] approach and the snpgdsIBD command in the SNPRelate R package[2]. These kinship estimates were provided to the pcair[3] command in the GENESIS R package[4] to perform principal components analysis while properly adjusting for relatedness within the data. We then used the pcrelate[5] command in the GENESIS R package to adjust the kinship estimates for the first four principal components.

The correlation between observed ε2 and ε4 genotypes and imputed genotypes at rs7412 and rs429358 were estimated with the cor.test function in R[6].

©2020 Blue EE et al. *JAMA Network Open*.

eTable 1. Sample Summary Table by ADGC Cohort

Cohort	Acronym	N_{cases}	$N_{controls}$	$N_{missing}$	N_{female}	%female
Adult Changes in Thought	ACT	527	1463	0	1135	57%
Adult Changes in Thought 2	ACT2	21	7	0	18	64%
NIA-funded AD Centers 1	ADC1	1537	0	0	835	54%
NIA-funded AD Centers 2	ADC2	653	143	0	427	54%
NIA-funded AD Centers 4	ADC4	284	364	0	393	61%
NIA-funded AD Centers 5	ADC5	270	494	0	466	61%
NIA-funded Centers 6	ADC6	213	326	0	339	63%
AD Neuroimaging Initiative	ADNI	77	62	0	58	42%
Biomarkers of Cognitive Decline among Normal Individuals	BIOCARD	6	112	0	72	61%
Chicago Health and Aging Project	CHAP	27	143	0	92	54%
Einstein Aging Study	EAS	9	141	0	62	41%
NIA Late-Onset AD Family Study	LOAD	1798	1568	0	2112	63%
Jacksonville Mayo Clinic	MAYO	590	960	0	839	54%
Oregon Health Sciences University	OHSU	128	143	0	159	59%
Rochester Mayo Clinic	RMayo	12	214	0	89	39%
Rush Religious Orders Study and Rush Memory Aging Study	ROSMAP	271	651	25	675	71%
Rush Religious Orders Study and Rush Memory Aging Study 2	ROSMAP2	49	73	0	93	76%
Texas Alzheimer's Research and Care Consortium	TARC1	275	169	0	280	63%

©2020 Blue EE et al. *JAMA Network Open*.

Translational Genomics Research Institute 2	TGEN2	665	359	0	604	59%
Saarland University	UKS	593	7	0	346	58%
University of Pittsburgh	UPITT	1254	827	0	1313	63%
Washington University St. Louis	WASHU	335	187	0	304	58%
Washington University St. Louis 2	WASHU2	37	94	0	65	50%
Washington Heights-Inwood Community Aging Project	WHICAP	73	559	0	391	62%

Abbreviations: N_{cases} = number of subjects affected by Alzheimer's disease, $N_{controls}$ = number of subjects not affected by Alzheimer's disease, $N_{missing}$ = number of subjects missing phenotype data, N_{female} = number of female subjects, AD: Alzheimer's Disease, NIA: National Institute on Aging. Cohort acronyms are identical to those provided by the ADGC in the downloaded data set. These data are accessible to the research community through the National Institute on Aging Genetics of Alzheimer's Disease Data Storage Site (https://www.niagads.org/resources/related-projects/alzheimers-disease-genetics-consortium-adgc-collection).

©2020 Blue EE et al. *JAMA Network Open*.

eTable 2. Evidence for Linkage Disequilibrium Among the *TOMM40*, *APOC1*, and *APOE* SNVs

	**rs2075650**	**rs4420638**	**rs7412**	**rs429358**
**rs2075650**	1	.30	< .20	.48
**rs4420638**	.50	1	< .20	.65
**rs7412**	.01	.02	1	< .20
**rs429358**	.63	.83	.02	1

Linkage disequilibrium is measured using r^2. The upper triangle reports estimates from the 1000 Genomes Europeans data, the lower triangle reports estimates from the ADGC data.

©2020 Blue EE et al. *JAMA Network Open*.

eTable 3. Evidence for Association Between rs8106922 With and Without *APOE* Adjustment or Stratification

model	SNP	N	AAF	OR	95% CI	***P***
**1**	rs8106922	18396	.35	.69	.65-.72	5.91E-50
**2**	rs8106922	18396	.35	1.03	.97-1.09	.40
**3**	rs8106922	8618	.51	1.02	.95-1.09	.63
**4**	rs8106922	1499	.02	2.11	.77-5.78	.15

Abbreviations: N = sample number, AAF = alternate allele frequency, OR = odds ratio, CI = confidence interval. Model 1: all samples, no *APOE* adjustment; model 2: all samples, adjust for *APOE* ε2 and ε4 allele counts; model 3: restricted to *APOE* ε3 homozygotes; model 4: restricted to *APOE* ε4 homozygotes.

©2020 Blue EE et al. *JAMA Network Open*.

eTable 4. Comparison of *APOE* ε2 and ε4 Genotypes With Imputed Genotypes at rs7412 and rs429358

	***APOE* ε2 vs. rs7412**				***APOE* ε4 vs. rs429358**			
**genotyping strategy**	N_{mis}	N_{total}	**r**	r^2	N_{mis}	N_{total}	**r**	r^2
Overall	444	35392	.88	.77	798	35306	.94	0.88
rs7412 and rs429358 SNPs	222	21412	.90	.81	431	20948	.95	0.90
Pyrosequencing or RFLP	109	7290	.84	.71	227	7408	.91	0.82
high-throughput sequencing	59	1708	.68	.47	46	1984	.88	0.78

N_{mis}: the number of discordant alleles mismatched between observed and imputed genotypes; N_{total}: the total number of alleles observed; r: the Pearson correlation coefficient, r^2: proportion of the variance in the imputed genotypes explained by the observed genotypes; RFLP: restriction fragment length polymorphism.

©2020 Blue EE et al. *JAMA Network Open*.

eFigure 1. Principal Components Analysis Results

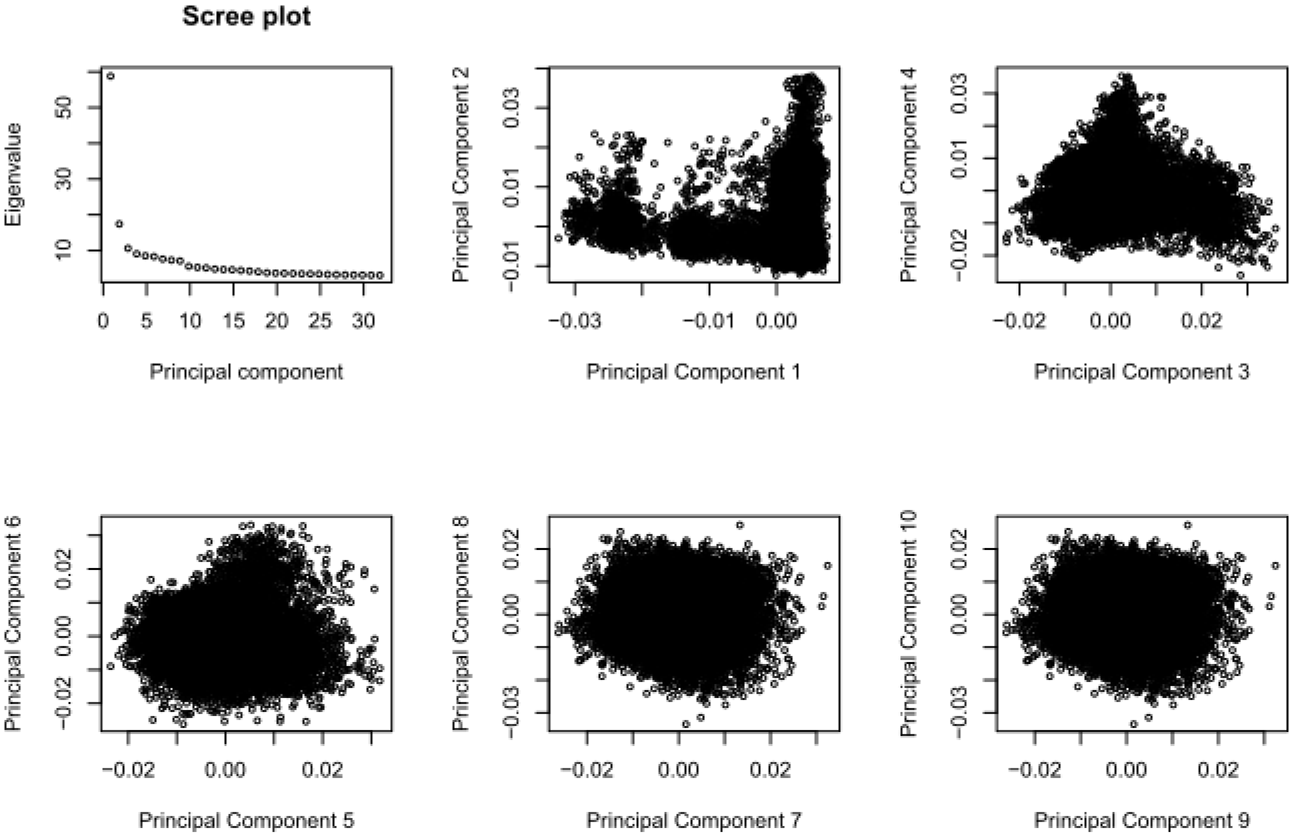

A.) A scree plot summarizes the eigenvalues for each principal component (PC) included as a covariate in the association tests. B.) Subject-specific values for PC1 vs. PC2. C.) Subject-specific values for PC3 vs. PC4. D.) Subject-specific values for PC5 vs. PC6. E.) Subject-specific values for PC7 vs. PC8. F.) Subject-specific values for PC9 vs. PC10.

©2020 Blue EE et al. *JAMA Network Open*.

eFigure 2. Difference Between Kinship Estimates Based on Genotypes for All Autosomes vs All Autosomes Except Chromosome 19

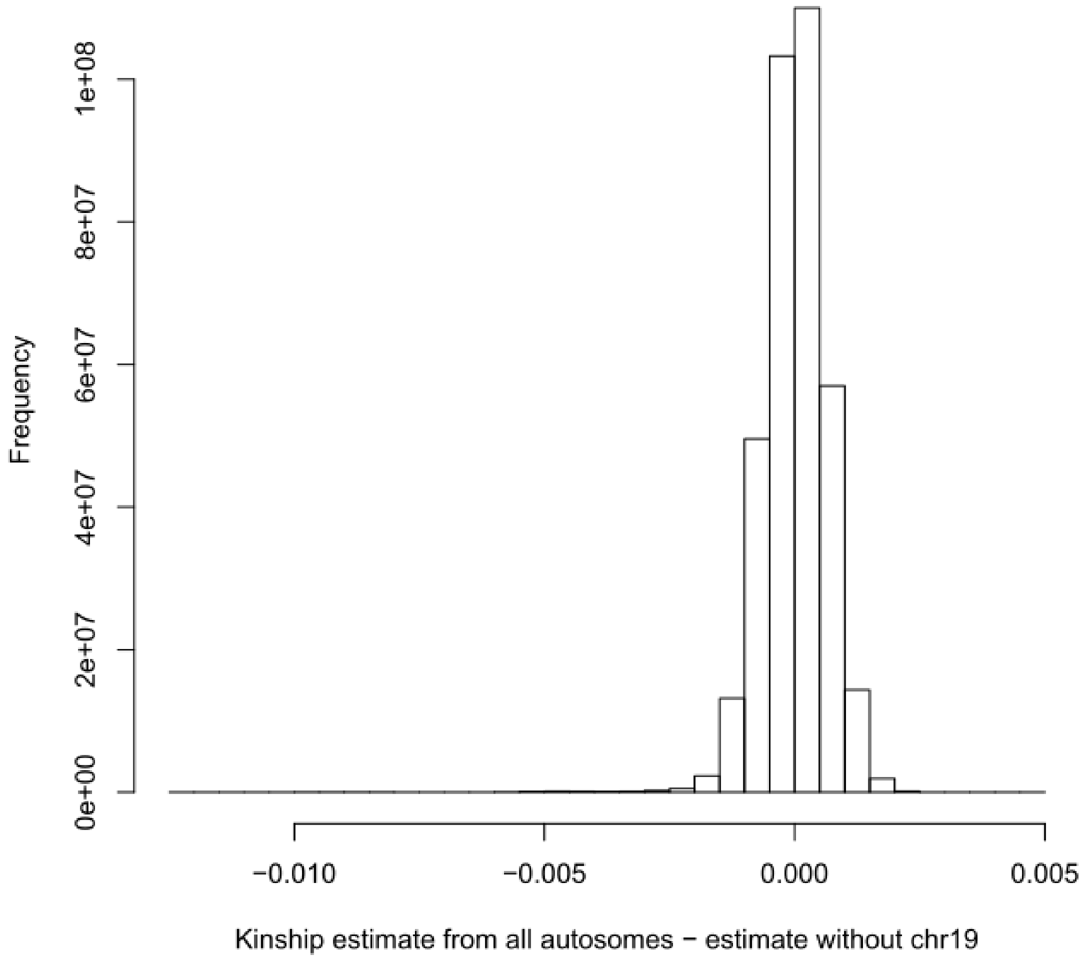

The x-axis is the numerical difference between the KING-robust pairwise kinship estimates including all autosomal variants with a minor allele frequency > 1% and a missing rate < 5% and the estimates including the same variant set excluding those on chromosome 19.

©2020 Blue EE et al. *JAMA Network Open*.

eFigure 3. Quantile-Quantile Plots of Genome-Wide Association Tests Under Each Analysis Model

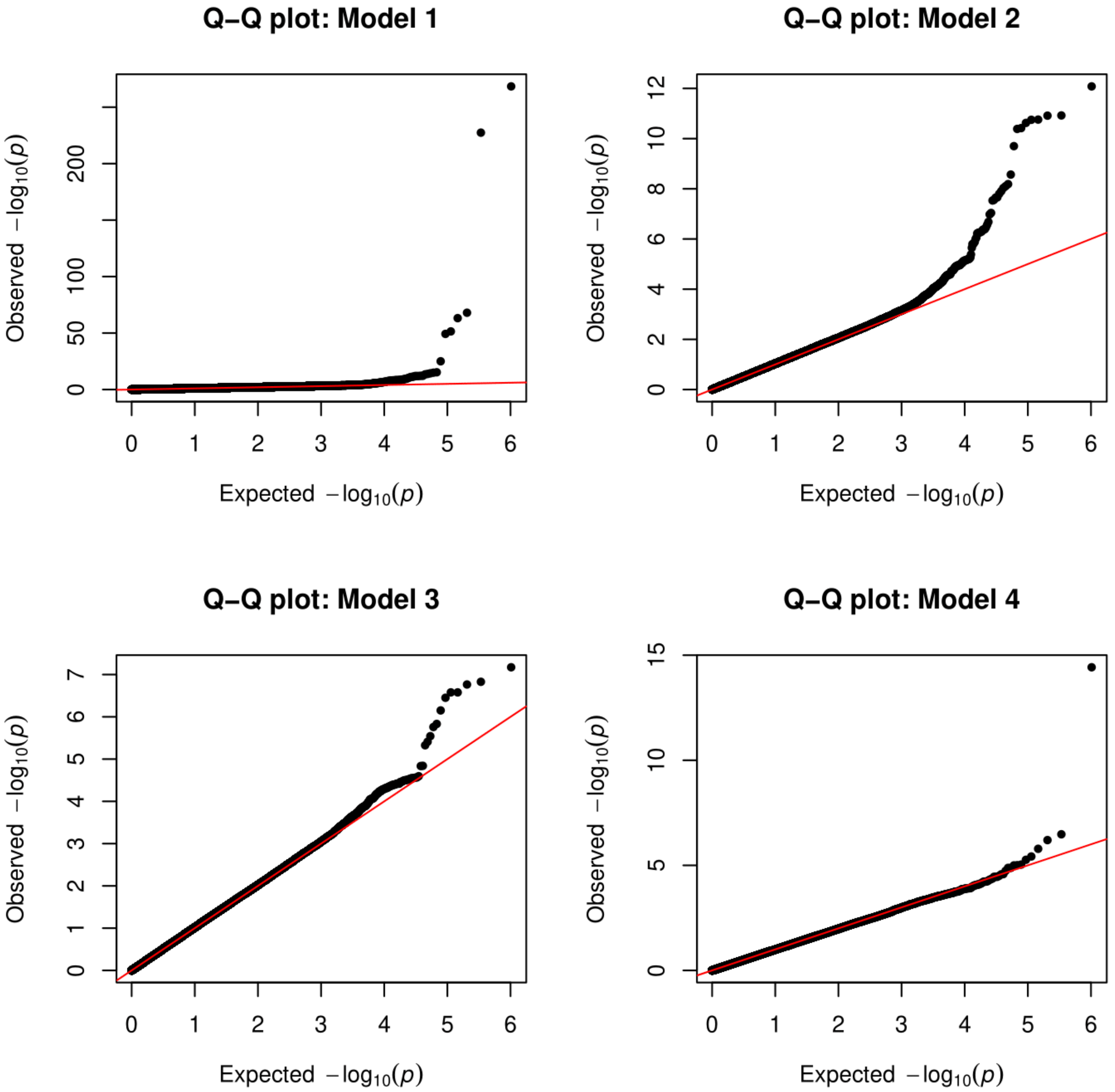

Q-Q: quantile-quantile; Model 1: all samples, no *APOE* adjustment; Model 2: all samples, adjust for *APOE* ε2 and ε4 allele counts; Model 3: restricted to *APOE* ε3 homozygotes; Model 4: restricted to *APOE* ε4 homozygotes; *p* = p-value.

©2020 Blue EE et al. *JAMA Network Open*.

References.

1. Manichaikul A, Mychaleckyj JC, Rich SS, Daly K, Sale M, Chen WM. Robust relationship inference in genome-wide association studies. *Bioinformatics.* 2010;26(22):2867-2873.
2. Zheng X, Levine D, Shen J, Gogarten SM, Laurie C, Weir BS. A high-performance computing toolset for relatedness and principal component analysis of SNP data. *Bioinformatics.* 2012;28(24):3326-3328.
3. Conomos MP, Miller MB, Thornton TA. Robust inference of population structure for ancestry prediction and correction of stratification in the presence of relatedness. *Genet Epidemiol.* 2015;39(4):276-293.
4. *GENESIS: GENetic EStimation and Inference in Structured samples (GENESIS): Statistical methods for analyzing genetic data from samples with population structure and/or relatedness. R package version 2.4.0.* [computer program]. 2016.
5. Conomos MP, Reiner AP, Weir BS, Thornton TA. Model-free Estimation of Recent Genetic Relatedness. *Am J Hum Genet.* 2016;98(1):127-148.
6. *R: A language and environment for statistical computing* [computer program]. Vienna, Austria: the R Foundation for Statistical Computing; 2011.

©2020 Blue EE et al. *JAMA Network Open*.
